# Respiratory event index underestimates severity of sleep apnea compared to apnea-hypopnea index

**DOI:** 10.1093/sleepadvances/zpad054

**Published:** 2023-12-22

**Authors:** Minna Pitkänen, Rajdeep Kumar Nath, Henri Korkalainen, Sami Nikkonen, Alaa Mahamid, Arie Oksenberg, Brett Duce, Juha Töyräs, Samu Kainulainen, Timo Leppänen

**Affiliations:** Department of Technical Physics, University of Eastern Finland, Kuopio, Finland; Department of Technical Physics, University of Eastern Finland, Kuopio, Finland; VTT Technical Research Centre of Finland Ltd, Kuopio, Finland; Department of Technical Physics, University of Eastern Finland, Kuopio, Finland; Diagnostic Imaging Center, Kuopio University Hospital, Kuopio, Finland; Department of Technical Physics, University of Eastern Finland, Kuopio, Finland; Diagnostic Imaging Center, Kuopio University Hospital, Kuopio, Finland; Sleep Disorders Unit, Loewenstein Hospital-Rehabilitation Center, Raanana, Israel; Sleep Disorders Unit, Loewenstein Hospital-Rehabilitation Center, Raanana, Israel; Sleep Disorders Centre, Department of Respiratory and Sleep Medicine, Princess Alexandra Hospital, Brisbane, Australia; Institute for Health and Biomedical Innovation, Queensland University of Technology, Brisbane, Australia; Department of Technical Physics, University of Eastern Finland, Kuopio, Finland; Science Service Center, Kuopio University Hospital, Kuopio, Finland; School of Electrical Engineering and Computer Science, The University of Queensland, Brisbane, Australia; Department of Technical Physics, University of Eastern Finland, Kuopio, Finland; Diagnostic Imaging Center, Kuopio University Hospital, Kuopio, Finland; Department of Technical Physics, University of Eastern Finland, Kuopio, Finland; Diagnostic Imaging Center, Kuopio University Hospital, Kuopio, Finland; School of Electrical Engineering and Computer Science, The University of Queensland, Brisbane, Australia

**Keywords:** OSA, sleep disordered breathing, home testing

## Abstract

Polygraphy (PG) is often used to diagnose obstructive sleep apnea (OSA). However, it does not use electroencephalography, and therefore cannot estimate sleep time or score arousals and related hypopneas. Consequently, the PG-derived respiratory event index (REI) differs from the polysomnography (PSG)-derived apnea-hypopnea index (AHI). In this study, we comprehensively analyzed the differences between AHI and REI. Conventional AHI and REI were calculated based on total sleep time (TST) and total analyzed time (TAT), respectively, from two different PSG datasets (*n* = 1561). Moreover, TAT-based AHI (AHI_TAT_) and TST-based REI (REI_TST_) were calculated. These indices were compared keeping AHI as the gold standard. The REI, AHI_TAT_, and REI_TST_ were significantly lower than AHI (*p* < 0.0001, *p* ≤ 0.002, and *p* ≤ 0.01, respectively). The total classification accuracy of OSA severity based on REI was 42.1% and 72.8% for two datasets. Based on AHI_TAT_, the accuracies were 68.4% and 85.9%, and based on REI_TST_, they were 65.9% and 88.5% compared to AHI. AHI was most correlated with REI_TST_ (*r* = 0.98 and *r* = 0.99 for the datasets) and least with REI (*r* = 0.92 and *r* = 0.97). Compared to AHI, REI had the largest mean absolute errors (13.9 and 6.7) and REI_TST_ the lowest (5.9 and 1.9). REI had the lowest sensitivities (42.1% and 72.8%) and specificities (80.7% and 90.9%) in both datasets. Based on these present results, REI underestimates AHI. Furthermore, these results indicate that arousal-related hypopneas are an important measure for accurately classifying OSA severity.

**Statement of Significance**

The diagnosis of sleep apnea is often based on polygraphy which does not allow the determination of arousals or total sleep time (TST) due to the lack of electroencephalography. This may lead to an underestimation of sleep apnea severity compared to the gold-standard polysomnography. However, this has not been previously studied in detail. We comprehensively examined the effect of TST and hypopneas followed by arousals on the severity of sleep apnea and found that both are crucial for the diagnosis. However, previously the traditional severity indices have been poorly correlated with symptoms, and therefore, further investigations are needed to link the present findings to the risk and prevalence of symptoms and comorbidities.

## Introduction

Obstructive sleep apnea (OSA) is one of the most prevalent sleep disorders affecting globally about 1 billion adults [1]. OSA is directly connected to several chronic life-threatening health conditions such as hypertension and coronary heart diseases [2]. Furthermore, indirect consequences of OSA include a decrease in the overall quality of life and an increased risk of work-related and road accidents due to OSA-related daytime sleepiness [3, 4]. Hence, proper diagnosis and timely treatment of OSA can potentially reduce the negative impacts of OSA and can save billions of dollars in healthcare costs [5]. The current diagnosis of OSA is mainly based on the number of respiratory events per hour of sleep, i.e. the apnea-hypopnea index (AHI), and involves a polysomnography (PSG) study as the gold standard. However, PSG study requires well-equipped sleep laboratory facilities and a complex sensor setup which limits the use of PSG in terms of scalability and cost.

Portable sleep monitoring, such as polygraphy (PG), provides a simpler and cheaper alternative to in-lab PSG studies [6]. However, portable systems usually lack electroencephalography (EEG) sensing modalities and thus, the estimation of the total sleep time (TST) and the scoring of hypopneas associated with arousals are not possible. Hence, the sleep apnea severity based on PG is estimated by utilizing the total analyzed time (TAT) or total bedtime instead of TST when calculating severity indices. Therefore, instead of the AHI, the severity index calculated using such a method is referred to as the respiratory event index (REI). REI is always less than or equal to the AHI underestimating the actual severity compared to that defined based on the AHI. The underestimation of the number of respiratory events and the overestimation of the sleep time can result in the misclassification of sleep apnea severity [7]. This can lead to misdiagnosis having a substantial effect on treatment decisions.

The fact that PG recordings lead to underestimation of sleep apnea severity is well known; however, the magnitude of the difference between PG- and PSG-based severity metrics is unknown and previous results are inconsistent to some extent. In adults with a high pretest likelihood of having moderate to severe OSA, the diagnostic performance of PG has been good in comparison with PSG, but the diagnostic accuracy decreases in patients with mild OSA [8]. Some previous studies analyzed the differences between AHI and REI by considering only the TAT instead of TST [9–13], whereas some other studies investigated the effect of arousals on the number of hypopneas, too [6, 14–20]. Based on these studies, there is a tendency for REI to underestimate AHI but not always the extent to affect diagnostic decisions. However, the differences between the values of AHI and REI have not been analyzed by considering the effect of arousals and the use of TST vs. TAT in detail.

In this study, we hypothesized that REI underestimates the severity of OSA, especially in mild and moderate cases, and that ignoring hypopneas associated with EEG-related arousals has a greater effect on the values of severity indices than inaccurate sleep time (i.e. TST vs. TAT). Hence, we aim to conduct a comprehensive analysis of the differences in OSA severity estimation obtained from AHI and REI which were calculated from the same full PSG data recorded on a single night. Furthermore, we also define and evaluate the performance of two new OSA severity measures: TAT-based AHI (AHI_TAT_) and TST-based REI (REI_TST_; variants of AHI and REI). To the best of our knowledge, this is the first study in which these two metrics are used for OSA severity classification and compared to conventional REI and AHI. This was done to evaluate whether inaccurate estimation of TST or exclusion of hypopneas associated with arousals has a greater effect on the values of severity indices and thus, treatment decisions.

## Materials and Methods

In this study, we retrospectively analyzed two separate datasets consisting of suspected OSA patients. The first dataset (dataset-1) consisted of 887 complete PSG recordings collected from suspected OSA patients during 2015–2017 at the Princess Alexandra Hospital (Brisbane, Australia). The second dataset (dataset-2) consisted of 937 complete PSG recordings collected during 2007–2009 at the Sleep Disorders Unit, Loewenstein Hospital—Rehabilitation Center (Raanana, Israel). The permission to use Australian and Israelian data has been granted by the Institutional Human Research Ethics Committee at the Princess Alexandra Hospital (permit numbers: HREC/16/QPAH/021 and LNR/2019/QMS/54313) and the Ethical Committee of the Loewenstein Hospital—Rehabilitation Center (permit number: 0006-17-LOE), respectively.

Dataset-1 PSGs were collected with Compumedics devices (Compumedics, Abbotsford, Australia) and analyzed with Compumedics Profusion 4.0 software (Build 410). The recording montage comprised an EEG (F4-M1, C4-M1, and O2-M1), left and right electrooculograms (recommended derivation: E1-M2 and E2-M2), a chin electromyogram (EMG; mental/submental positioning), a modified lead II electrocardiograph, nasal pressure, an oronasal thermocouple, body position, thoracic and abdominal effort (inductive plethysmography), pulse oximetry (Nonin Xpod 3011), right and left leg movement (anterior tibialis EMG), and sound pressure (dBA meter).

Dataset-2 was collected with Rembrandt Manager System (MedCare Co, Amsterdam, The Netherlands). The patients had complained about daytime sleepiness, but no questionnaires were filled. The recorded signals varied slightly between patients. Typically, recordings comprised an EEG with electrodes C4, C3, Pz, A1, and A2, right and left electrooculogram, a chin EMG, electrocardiograph, airflow thermistor, airflow pressure, body position, chest and abdomen respiratory belts, pulse oximetry, right and left leg movement (anterior tibialis), snore pressure, and audio signal.

The respiratory events and the sleep stages for dataset-1 and dataset-2 were scored manually by expert sleep technicians based on the American Academy of Sleep Medicine (AASM) scoring manual 2012 [21] and 2007 [22], respectively. The difference between the scorings is in the hypopnea scoring rule. The hypopneas were scored when the airflow signal dropped by at least 30% for at least 10 seconds and they were associated with at least 3% (dataset-1) or 4% (dataset-2) oxygen desaturation or an arousal.

TST was defined as the total time the patient slept based on EEG. The time duration between the manually marked lights off and lights on times was used as TAT. In both datasets, recordings with TAT less than 4 hours or without the information on the TAT were excluded from the analysis. Hence, the final analysis consisted of 636 patients from dataset-1 and 925 patients from dataset-2.

Conventional AHI and REI were calculated based on TST and TAT, respectively. AHI_TAT_ was calculated by substituting TST with TAT for AHI. REI_TST_ was calculated by substituting TAT with TST for REI (Table 1). The hypopneas included in the REI calculation were associated with oxygen desaturations starting at the latest 30 seconds after hypopnea ended. The AHI included all hypopneas scored.

**Table 1.** Severity Indices Utilized in This Study

Severity index	Definition
AHI	$=\frac{no.\;of\;all\;apneas+no.\;of\;all\;hypopneas\;\;\;}{TST}$
REI	$=\frac{no.\;\;of\;all\;apneas+no.\;\;of\;hypopneas\;followed\;by\;desaturations\;only}{TAT}$
AHI_TAT_	$=\frac{no.\;of\;all\;apneas+no.\;of\;all\;hypopneas\;\;\;}{TAT}$
REI_TST_	$=\frac{no.\;of\;all\;apneas+no.\;\;of\;hypopneas\;followed\;by\;desaturations\;only}{TST}$

TAT is the time between lights off and lights on. TST is the total time the patient slept based on EEG recording. Scoring of hypopneas and apneas was based on AASM scoring manuals 2007 (dataset-2) and 2012 (dataset-1). The hypopneas were scored when the airflow signal dropped by at least 30% for at least 10 seconds and they were associated with at least 3% (dataset-1) or 4% (dataset-2) oxygen desaturation or an arousal.

Abbreviations: AHI, apnea-hypopnea index; REI, respiratory event index; TAT, total analyzed time; TST, total sleep time.

The agreement of REI, AHI_TAT_, and REI_TST_ with AHI was evaluated using linear regression. The Pearson correlation coefficient, and the slope of the fitted regression line between REI, AHI_TAT_, and REI_TST_, and the AHI were calculated as well as the mean absolute errors (MAEs), maximum errors, and absolute percentage errors of REI, AHI_TAT_, and REI_TST_ with AHI. The statistical significance of differences between the indices was evaluated using the Mann–Whitney *U* (MWU) test.

The sleep apnea severity (no-OSA, mild OSA, moderate OSA, and severe OSA) was defined based on the AHI and used as the ground truth. The AHI-based OSA severity was then compared to that defined by REI, AHI_TAT_, and REI_TST_. The cut-off values of 5–15–30 events/hour were used for mild, moderate, and severe OSA, respectively. The consistency of OSA-severities based on REI, AHI_TAT_, and REI_TST_ with the AHI-based severity classification was analyzed using classification metrics accuracy, F1-score, sensitivity, and specificity, which were estimated by macro averaging, i.e. first calculating the metrics for all four OSA severity groups and then calculating the average of each metric [23, 24]. Thus, the macro average is the arithmetic mean of the individual severity groups related to accuracy, F1-score, sensitivity, and specificity. Another way would have been to calculate the micro average, i.e. to calculate a value for each metric from pooled data instead of calculating the metrics separately for each severity group then taking the average. However, macro averaging should be used when there are unequal number of patients in different groups as the method gives equal weight for each group. Furthermore, intra-class correlation (ICC) analysis was done to compare the different OSA severity groups based on the classifications by AHI, REI, AHI_TAT_, and REI_TST_.

## Results

**Figure 1.**
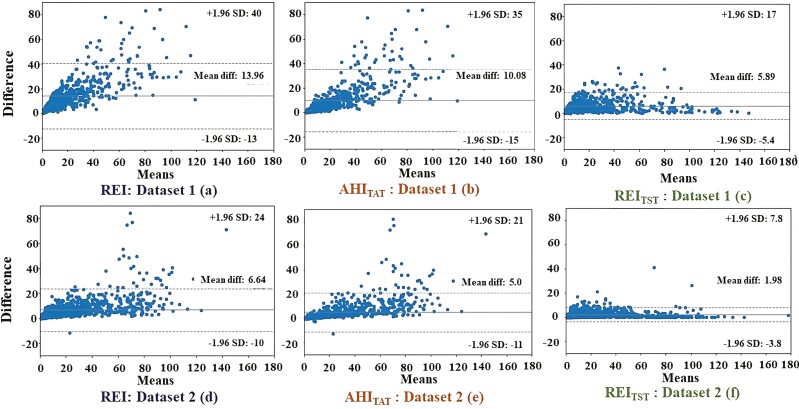
Bland–Altman plots for dataset 1 (a–c) and dataset 2 (d–f). Abbreviations: AHI, apnea-hypopnea index; AHI_TAT_, AHI based on total analyzed time; REI, respiratory event index; REI_TST_, REI based on total sleep time.

Clinical characteristics of the patients are shown in Table 2 and median respiratory event characteristics for the patients are represented in Tables 3 and 4. In both datasets, the REI was significantly lower than the AHI in all OSA severity groups when the groups were defined based on the AHI (MWU *p* < 0.0001). Similarly, AHI_TAT_ and REI_TST_ were also significantly lower compared to the AHI (MWU *p* < 0.0001 in dataset-1, *p* = 0.002 and *p* = 0.01 in dataset-2, respectively) in all OSA severity groups. The Pearson correlation coefficient (*r*) and the slope (*b*) of the fitted regression line between REI, AHI_TAT_, and REI_TST_, and the AHI were found to be the highest for REI_TST_ and lowest for REI in both the datasets (Table 5). In both datasets, REI had the highest MAE values and REI_TST_ had the lowest MAE values. A similar trend was observed for maximum errors in both datasets. When comparing parameter values to the AHI, REI had the greatest maximum errors (84.4 events/hour for dataset-1 and 84.3 events/hour for dataset-2) and REI_TST_ had the smallest maximum errors (37.3 events/hour for dataset-1 and 41.2 events/hour for dataset-2). The ICCs between the OSA severity groups, estimated by using the severity indices AHI, REI, AHI_TAT_, and REI_TST_, were 0.89 and 0.97 for dataset-1 and dataset-2, respectively. Bland–Altman plots are represented in Figure 1.

**Table 2.** Clinical Characteristics of the Patients

	Dataset-1				Dataset-2			
	*n*	Age (years)	Male (%)	BMI (kg/m^2^)	*n*	Age (years)	Male (%)	BMI (kg/m^2^)
Population	636	54.1 ± 14.4	54.6	36.0 ± 9.7	925	47.3 ± 16.1	75.1	30.6 ± 7.3
No-OSA (AHI < 5)	90	45.3 ± 15.1	33.3	31.9 ± 8.7	209	36.7 ± 16.5	58.4	26.2 ± 5.8
Mild (5 ≤ AHI < 15)	172	54.3 ± 13.4	43.0	34.5 ± 9.4	220	47.6 ± 14.7	70.9	29.9 ± 8.2
Moderate (15 ≤ AHI < 30)	156	55.5 ± 13.4	59.5	35.9 ± 8.3	187	51.7 ± 14.4	81.8	31.3 ± 5.5
Severe (AHI ≥ 30)	218	56.6 ± 14.2	68.8	38.9 ± 10.3	309	51.7 ± 14.4	85.4	33.8 ± 6.6

The values are presented as mean ± standard deviation for continuous variables.

Dataset-1 and dataset-2 are collected in Australia and Israel, respectively.

Abbreviations: AHI, apnea-hypopnea index; BMI, body mass index; *n*, number of patients; OSA, obstructive sleep apnea.

**Table 3.** Respiratory Event Characteristics for the Patients in the Dataset-1

Dataset-1					
	The whole population	No-OSA (AHI < 5)	Mild OSA (5 ≤ AHI < 15)	Moderate OSA (15 ≤ AHI < 30)	Severe OSA (AHI ≥ 30)
*n*	636	90	172	156	218
AI (events/hr)	1.4 (0.0–142.3)	0.2 (0.0–1.7)	0.4 (0.0–8.8)	1.8 (0.0–22.6)	10.3 (0.0–142.3)
HI (events/hr)	15.4 (0.0–122.2)	2.4 (0.0–4.9)	8.6 (0.0–14.9)	18.0 (3.5–28.8)	36.0 (4.9–122.2)
H_D_I (events/hr)	9.1 (0.0–118.6)	0.5 (0.0–4.1)	3.8 (0.2–11.7)	11.5 (0.0–26.0)	28.0 (1.4–118.6)
H_A_I (events/hr)	4.1 (0.0–37.3)	1.2 (0.0–3.8)	3.9 (0.0–13.8)	6.3 (0.0–24.6)	5.5 (0.0–37.3)
TST (hr)	5.1 (0.5–8.3)	5.7 (1.8–7.6)	5.4 (1.2–8.3)	5.2 (0.5–8.0)	4.8 (0.8–7.8)
TAT (hr)	7.4 (4.9–9.7)	7.4 (5.9–8.9)	7.4 (5.8–9.2)	7.4 (4.9–9.3)	7.3 (5.0–9.7)
AHI (events/hr)	19.4 (0.2–147.2)	2.8 (0.2–4.9)	9.6 (5.0–15.0)	21.6 (15.0–30.0)	51.6 (30.1–147.2)
REI (events/hr)	8.4 (0.1–113.2)	0.6 (0.1–3.2)	3.2 (0.2–12.1)	9.9 (0.2–24.1)	27.9 (6.1–113.2)
AHI_TAT_ (events/hr)	13.1 (0.1–114.6)	1.9 (0.1–4.2)	6.7 (2.0–12.9)	15.3 (1.6–25.9)	32.7 (8.3–114.6)
REI_TST_ (events/hr)	12.6 (0.2–147.2)	1.0 (0.2–4.1)	5.1 (0.3–13.3)	14.3 (1.1–27.6)	42.2 (9.5–147.2)

The OSA severity groups were formed based on the AHI calculated from in-lab PSG. The values are presented as median (range).

Abbreviations: AHI, apnea-hypopnea index; AHI_TAT_, AHI based on total analyzed time; AI, apnea index; H_A_I, hypopnea index including only hypopneas followed by arousal; H_D_I, hypopnea index including only hypopneas followed by desaturation; HI, hypopnea index; hr, hour; *n*, number of patients; OSA, obstructive sleep apnea; PSG, polysomnography; REI, respiratory event index; REI_TST_, REI based on total sleep time; TAT, total analyzed time; TST, total sleep time.

**Table 4.** Respiratory Event Characteristics for the Patients in the Dataset-2

Dataset-2					
	The whole population	No-OSA (AHI < 5)	Mild OSA (5 ≤ AHI < 15)	Moderate OSA (15 ≤ AHI < 30)	Severe OSA (AHI ≥ 30)
*n*	925	209	220	187	309
AI (events/hr)	2.2 (0.0–104.2)	0.2 (0.0–2.6)	1.1 (0.0–11.6)	4.1 (0.0–22.2)	19.0 (0.0–140.2)
HI (events/hr)	11.8 (0.0–112.8)	1.7 (0.0–4.9)	7.8 (0.4–14.7)	16.2 (6.0–28.9)	31.3 (1.8–112.8)
H_D_I (events/hr)	8.9 (0.0–112.6)	1.0 (0.0–4.7)	5.7 (0.0–13.3)	13.8 (3.5–28.4)	29.2 (1.5–112.6)
H_A_I (events/hr)	0.9 (0.0–41.2)	0.3 (0.0–2.8)	1.3 (0.0–10.3)	2.2 (0.0–17.1)	0.9 (0.0–41.2)
TST (hr)	6.0 (1.9–13.0)	6.0 (2.4–7.4)	6.1 (2.7–9.3)	6.0 (2.8–13.0)	5.8 (1.9–9.1)
TAT (hr)	6.9 (4.4–9.6)	6.9 (4.4–8.7)	7.0 (4.7–8.8)	7.0 (5.3–8.0)	6.9 (5.4–9.6)
AHI (events/hr)	17.1 (0.3–178.4)	2.2 (0.3–5.0)	9.5 (5.0–14.8)	21.0 (15.0–30.0)	37.1 (30.3–178.4)
REI (events/hr)	12.1 (0.0–120.9)	1.3 (0.0–4.3)	6.5 (0.0–13.4)	14.7 (0.7–28.5)	44.1 (11.4–120.9)
AHI_TAT_ (events/hr)	14.4 (0.3–121.5)	1.8 (0.3–4.6)	8.3 (3.1–13.8)	17.4 (8.6–29.3)	46.4 (11.7–121.5)
REI_TST_ (events/hr)	14.2 (0.0–176.6)	1.6 (0.0–4.8)	7.7 (0.0–14.4)	17.8 (0.8–29.1)	55.9 (14.6–176.6)

The OSA severity groups were formed based on the AHI calculated from in-lab PSG. The values are presented as median (range).

Abbreviations: AHI, apnea-hypopnea index; AHI_TAT_, AHI based on total analyzed time; AI, apnea index; H_A_I, hypopnea index including only hypopneas followed by arousal; H_D_I, hypopnea index including only hypopneas followed by desaturation; HI, hypopnea index; hr, hour; *n*, number of patients; OSA, obstructive sleep apnea; PSG, polysomnography; REI, respiratory event index; REI_TST_, REI based on total sleep time; TAT, total analyzed time; TST, total sleep time.

**Table 5.** Mean Absolute Error (MAE), Maximum Error, and Pearson Correlation Coefficient (*r*) for REI, AHI_TAT_, and REI_TST_ with AHI as the Ground Truth

	Dataset-1			Dataset-2		
	MAE	Max error	*r*	MAE	Max error	*r*
REI	13.9	84.4	0.92	6.7	84.3	0.97
AHI_TAT_	10.1	83.8	0.93	5.0	82.4	0.97
REI_TST_	5.9	37.3	0.98	1.9	41.2	0.99

Abbreviations: AHI, apnea-hypopnea index; AHI_TAT_, AHI based on total analyzed time; MAE, mean absolute error; *r*, Pearson correlation coefficient; REI, respiratory event index; REI_TST_, REI based on total sleep time; TAT, total analyzed time; TST, total sleep time.

**Figure 2.**
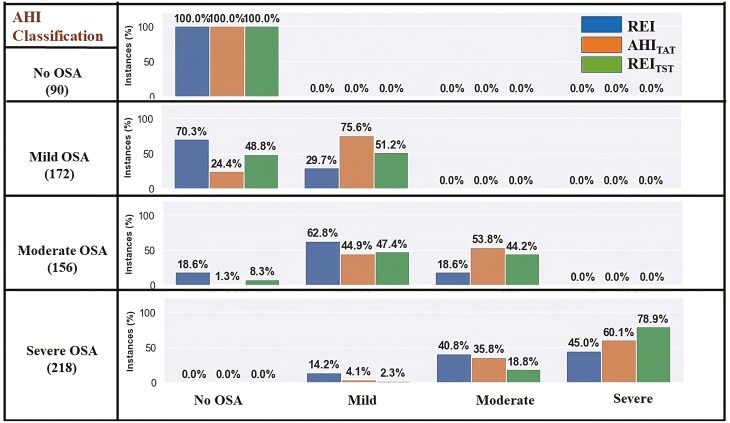
Illustration of misclassifications resulting from the use of different OSA severity metrics (REI, AHI_TAT_, and REI_TST_) within OSA severity groups formed based on the AHI calculated from full PSG (dataset-1). Abbreviations: AHI, apnea-hypopnea index; AHI_TAT_, AHI based on total analyzed time; OSA, obstructive sleep apnea; PSG, polysomnography; REI, respiratory event index; REI_TST_, REI based on total sleep time.

**Figure 3.**
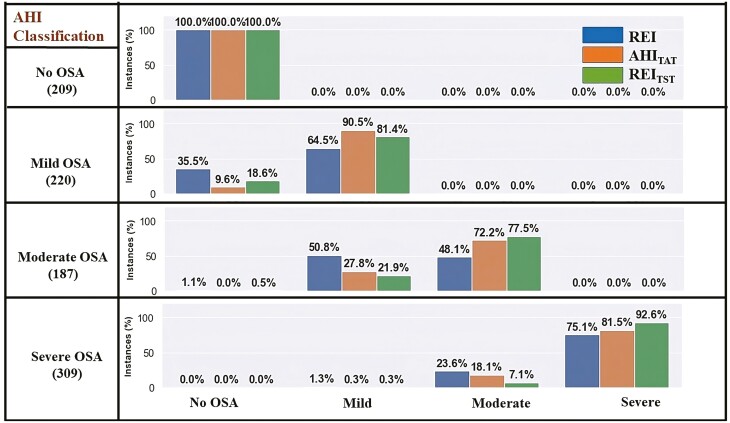
Illustration of misclassifications resulting from the use of different OSA severity metrics (REI, AHI_TAT_, and REI_TST_) within OSA severity groups formed based on the AHI calculated from full PSG (dataset-2). Abbreviations: AHI, apnea-hypopnea index; AHI_TAT_, AHI based on total analyzed time; OSA, obstructive sleep apnea; PSG, polysomnography; REI, respiratory event index; REI_TST_, REI based on total sleep time.

The overall accuracies of OSA severity classification (ground truth obtained using the AHI) using REI were 42.1% and 72.8% for dataset-1 and dataset-2, respectively. The percentages of correct classifications of OSA severity in mild, moderate, and severe OSA groups based on REI were low in both datasets (Figures 2 and 3). For example, the accuracies of REI in correctly classifying patients to have severe OSA were 45.0% and 75.1% in datasets 1 and 2, respectively. The classification accuracies were the lowest in cases of moderate OSA in both datasets (18.6% in dataset-1 and 48.1% in dataset-2). In addition, there were several cases when patients were severely underdiagnosed by utilizing REI. For example, 18.6% of the patients who were diagnosed to have moderate OSA by using the AHI were wrongly classified as having no-OSA in dataset-1. Similarly, in dataset-1, 14.2% of the patients were wrongly classified as having mild OSA when in fact, those patients belonged to the severe OSA group. However, such a variation was much smaller in dataset-2. Although the percentage of misclassification using AHI_TAT_ and REI_TST_ was lower compared to REI, there still were severely underdiagnosed patients. For example, in dataset-1, 1.3% and 8.3% of the patients with moderate OSA were misclassified as having no-OSA using AHI_TAT_ and REI_TST_, respectively. Similarly, 4.1% (using AHI_TAT_) and 2.3% (using REI_TST_) of the patients were incorrectly diagnosed as having mild OSA, when in fact those patients belonged to the severe OSA group.

Analysis of the performance metrics of individual OSA severity groups showed that the F1-score of mild and moderate severity groups was lower compared to the severe group in both datasets using REI as the severity index. However, this could be because there is no upper limit in the severe group (AHI ≥ 30). For example, the F1-scores for the mild and moderate OSA groups were 29.0% and 21.2%, whereas for the severe OSA group it was 62.0% in the dataset-1. Similarly, the F1-scores for the mild and moderate OSA groups in the dataset-2 were 61.6% and 51.4%, but that of the severe OSA group was 85.8%. Macro averaged F1-scores and overall accuracies are presented in Table 6.

**Table 6.** Macro Averaged Sensitivity, Specificity, F1-Score, and Overall Accuracy Values for REI, AHI_TAT_, and REI_TST_

	Dataset-1				Dataset-2			
	Sensitivity	Specificity	F1-score	Overall accuracy	Sensitivity	Specificity	F1-score	Overall accuracy
REI	42.1%	80.7%	41.7%	42.1%	72.8%	90.9%	70.4%	72.8%
AHI_TAT_	68.4%	89.5%	69.1%	68.4%	85.9%	95.3%	85.2%	85.9%
REI_TST_	65.9%	88.6%	64.2%	65.9%	88.5%	96.2%	87.5%	88.5%

The values are based on classifying a patient in the OSA severity groups (no-OSA, mild OSA, moderate OSA, and severe OSA) using REI, AHI_TAT_, and REI_TST_ with AHI as the ground truth.

Abbreviations: AHI, apnea-hypopnea index; AHI_TAT_, AHI based on total analyzed time; OSA, obstructive sleep apnea; REI, respiratory event index; REI_TST_, REI based on total sleep time; TAT, total analyzed time; TST, total sleep time.

The macro averaged sensitivities and specificities to classify patients into the OSA severity groups for REI, AHI_TAT_, and REI_TST_ are presented in Table 6. In both datasets, REI, AHI_TAT_, and REI_TST_ had higher specificity than sensitivity. AHI_TAT_ had the highest sensitivity (68.4%) and specificity (89.5%) in dataset-1, whereas in dataset-2, REI_TST_ had the highest sensitivity (88.5%) and specificity (96.2%). REI had the lowest sensitivity (42.1% and 72.8%) and specificity (80.7% and 90.9%) in both datasets.

## Discussion

In this study, we conducted a detailed analysis of differences between AHI and REI for OSA severity classification and introduced and evaluated the performance of two novel OSA severity metrics AHI_TAT_ and REI_TST_. The influence of TST and arousals in hypopnea scoring on severity assessment has never been investigated in such depth. Consideration of arousals and TST affects often-used home testing, as neither of those are recorded in PG. In our analysis, we directly addressed the fact that the information on hypopneas followed only by an arousal, as well as information on TST, is missing from the calculation of REI, which contributes to the underestimation of OSA severity. Our study’s findings and analysis were based on 1561 PSGs from two separate datasets. In both datasets, we found substantial discrepancies between REI and AHI across all OSA severity categories indicating that REI greatly underestimates OSA severity. Our research also revealed that the degree to which REI underestimates the true severity of OSA is alarming with more than 50% of the patients classified into the less severe OSA group in dataset-1 and more than 20% in dataset-2 (Figures 2 and 3). Moreover, some individuals with moderate OSA or severe OSA were classified into no-OSA or mild OSA groups respectively using REI as the severity index. This suggests the likelihood of severe underdiagnosis, which can result in large disparities in OSA care techniques resulting to unfavorable outcomes.

However, it should be noted that the AHI has been found to be poorly connected to OSA-associated symptoms (e.g. daytime sleepiness) and a variety of other health effects [25]. Therefore, it can be speculated whether REI underestimates the true severity of OSA or whether the AHI overestimates it. Further studies are needed to link both measurements to the incidence and prevalence of symptoms and comorbidities.

We also performed analyses on two alternative severity indices for OSA severity classification, AHI_TAT_ and REI_TST_ (variants of AHI and REI, respectively), and found that information on both TST and hypopneas followed solely by an arousal are critical for correct OSA severity classification. Our findings revealed that utilizing REI_TST_ as the severity measure resulted in serious underdiagnosis, with some patients with moderate OSA classified as having no-OSA and others with severe OSA were categorized as having mild OSA (Figure 2). AHI_TAT_ resulted in a higher proportion of misclassification for the severe OSA groups than REI_TST_, whereas REI_TST_ resulted in a higher percentage of misclassification for the mild OSA group. This supports the notion that when determining the true severity of OSA, correct detection of arousals (the ability to score hypopneas associated only with arousals) is more important than an accurate calculation of sleep time in mild OSA, whereas in severe OSA patients, there are fewer hypopneas followed by only an arousal than in mild and moderate OSA patients and hence, correct sleep time determination is important. The misclassification of AHI_TAT_ and REI_TST_ was roughly the same in the moderate group. Given the significant proportion of patients misclassified with both indices, information on both, arousal-related hypopneas and TST, is critical for accurately identifying OSA severities, particularly in moderate OSA patients. This discovery is critical and should be considered when developing new ambulatory monitoring devices that do not include EEG signals and algorithms for them; the surrogate signal should have enough information to allow accurate identification of arousals and assessment of sleep stages.

The findings of this investigation confirm the notion that AHI differs significantly from REI, at least when calculated using PSG data. Utilizing other thresholds for OSA severity classification rather than the typical 5–15–30 events/hour thresholds could lessen the possibilities of misclassification of OSA severity when utilizing REI. Previously, new thresholds based on mortality risk were proposed [26], raising questions regarding the validity of the existing thresholds.

The outcomes of this study reinforce the notion that it is critical to record EEG during PG or to be able to accurately estimate sleep stages and detect arousals from surrogate signals. Monitoring EEG signals necessitates a time-consuming sensor setup, which is a substantial impediment. Current research trends favor employing signal analysis and artificial intelligence methods to estimate TST, sleep stages, and arousals from non-EEG data such as photoplethysmogram (PPG) [27, 28] or heart rate [20, 29]. Incorporating such sophisticated signal analysis algorithms into clinics would be a feasible alternative to using EEG readings to determine sleep time, sleep stages, and arousals. For example, Vat et al. [28] studied whether the pulse wave amplitude (PWA) decreases from PPG could be utilized as a substitute for EEG-arousal in assessing hypopneas. They discovered that the inclusion of surrogate arousal in the hypopnea scoring increased sensitivity solely for identifying severe OSA participants but did not appear to significantly improve the PG overall accuracy. They also discovered a significant although modest correlation between PWA declines and EEG arousals. According to Mayer et al. [20], heart rate acceleration (HRa) and pulse transit time decrease may be utilized as surrogates for EEG arousals to assess hypopneas in PG at least in a mild and moderate OSA groups. Lachapelle et al. [29] carried out another study that used HRa in PG-based hypopnea scoring. They demonstrated that using HRa enhanced diagnostic agreement, clinical decision-making, and reduced the need for additional PSG testing. However, accurate detection of sleep stages and arousals from PPG or heart rate is still difficult and requires additional investigation. Even EEG-based arousal detection is problematic and manual scoring of arousals by expert scorers has substantial inter-scorer disagreement      [30]. As a result, it is critical to conduct considerable research on developing accurate models for estimating arousals, sleep stages, and sleep time utilizing minimally obtrusive methods.

This study has some limitations. Although this study included a large cohort of patients from two distinct sleep centers, the analyzed data only comprised a single-night full-PSG study and not actual PG or home recordings. Patients can sleep better at home than in a controlled lab setting especially when using a simple PG system, which could have influenced the results slightly. The PSG electrodes and laboratory environment might result in low sleep efficiency, and therefore direct comparison to home recordings cannot be made. However, utilizing PSG data instead of PG could have improved the quality of data used to evaluate the number of apneas and hypopneas. For example, in-lab PSG studies are likely to produce superior signal quality than home recordings, which can affect the fraction of actual respiratory events accurately detected. As a result, the actual difference between AHI and REI may be substantially greater. However, because the goal of this study was to compare REI, the variants of REI and AHI, and the gold standard OSA diagnostic metric (AHI), the present datasets were judged to be appropriate for this study. We would not have been able to study the impact of arousals and TST on the severity indices if we had used PG data that lacked EEG.

Another limitation is that different versions of the AASM manual were used when manually scoring the recordings in the two sleep centers. Previous studies have reported that the scoring based on AASM manual version 2007 leads to lower AHI than the scoring based on the 2012 version [31–33]. Because the hypopnea score definitions differ, the severity indices such as REI may have been influenced in this study. However, we consider this as a strength of this study, as the aim was to investigate the differences between different severity indices. Therefore, we can conclude that the scoring rules used have also an effect on the disparity between different severity measures—AASM 2007 scoring rules resulted in more consistent severity index values. However, disparities in the scorings might be attributable to other factors, such as time and effort of the scorer or center-specific conventions leading to inter-scorer variability [34, 35]. Furthermore, the lights off/on times were determined manually by the sleep laboratory staff, which might considerably differ from the TST. In the dataset-2, the median difference between TAT and TST (0.9 hours) was remarkably lower compared to the median difference of 2.1 hours between TAT and TST in the dataset-1, which may explain the larger REI-based severity misclassification in dataset-1 compared to dataset-2. Significant differences between TST and TAT have been found in home recordings, too [36].

The datasets used in this study differ slightly based on the patient characteristics, such as the average age and the proportion of male patients, which might have influenced the results. The dataset-1 consisted of older and more obese people and included more women than the dataset-2. Moreover, the dataset-2 had more people with no-OSA. In addition, we did not have access to all patient characteristics, such as smoking and comorbidities, on both datasets and therefore they have not been considered in the analysis. As the sample size is high and the results consistent, we consider that the differences in the datasets do not affect the conclusion.

In conclusion, to the best of our knowledge, this is the first study that has compared the effect of arousals and TST on the OSA severity estimation and introduced and evaluated two novel OSA severity metrics AHI_TAT_ and REI_TST_. REI can significantly underestimate the true severity of OSA and should be used with caution as a substitute for the PSG-based AHI, particularly when deciding on therapy courses. Our findings emphasize the need for measuring EEG during PG or at the least, the estimation of TST and arousals should be done from non-EEG signals, for example, by using sophisticated signal analysis and machine learning methods. Establishing unique norms and recommendations for conducting PG could be valuable in the future for boosting its dependability. Furthermore, attempts to develop alternative cost-effective and automated approaches for the detection of OSA should be among the clinical sleep research community’s top goals.

## Data Availability

The data include medical records and personal information and therefore the data can only be shared within the confinements of the Australian and Israeli legislation and ethical conventions. Reasonable requests considering data sharing will be individually assessed.
